# Physiological and Molecular Adaptation of the Ahuehuete (*Taxodium mucronatum* Ten.) to Waterlogging

**DOI:** 10.3390/plants14213295

**Published:** 2025-10-29

**Authors:** Yunpeng Gao, Dezong Sui, Shizheng Shi, Jingwen Zou, Shuai Wang, Liyong Sun, Cong Lei, Shuxian Li, Hongling Wang

**Affiliations:** 1 Jiangsu Academy of Forestry, Nanjing 211153, China; gaoyp5ai9@163.com (Y.G.); suidezong@163.com (D.S.); shshzn@163.com (S.S.); zjw18262915611@163.com (J.Z.); ws15195643260@163.com (S.W.); 2 Co-Innovation Center for Sustainable Forestry in Southern China, College of Forestry, Nanjing Forestry University, Nanjing 210037, China; sunly@njfu.edu.cn (L.S.); leicong@njfu.edu.cn (C.L.); shuxianli@njfu.com.cn (S.L.)

**Keywords:** *Taxodium mucronatum* Ten., transcriptomic analysis, stress response, osmoprotectants, antioxidant enzyme system, MAPK signal pathway

## Abstract

Ahuehuete (*Taxodium mucronatum* Ten.) is a riparian tree species of significant ecological, cultural, and economic importance, demonstrating remarkable tolerance to prolonged flooding. However, the underlying mechanism of waterlogging adaptation remains unknown. In this study, we determined the physiological traits of the Ahuehuete leaves at 0, 15, 30, and 60 d under waterlogging conditions. The results showed that no significant difference in MDA content occurred between the Ahuehuete leaves subjected to waterlogging and those under well-watered (CK) conditions. In contrast, the contents of osmoprotectants (soluble sugar, soluble protein, and proline) and the activities of antioxidant enzymes (SOD, POD, and CAT) exhibited similar change trends under both waterlogging and CK conditions, despite minor quantitative differences between the two groups. Subsequent comparative transcriptome analysis was performed to investigate the transcriptional characteristics. A total of 3687 DEGs were expressed in all comparisons throughout the waterlogging process, while 2873, 4617, and 2710 DEGs were comparison group specific. KEGG enrichment analysis revealed that DEGs were enriched in various metabolic pathways, such as Plant hormone signal transduction (ko04075), MAPK signaling pathway-plant (ko04016), ABC transporter (ko02010), and Nitrogen metabolism (ko00910). WGCNA also identified key modules associated with physiological traits, simultaneously emphasizing the importance of plant hormone signal transduction and MAPK signal cascade. Overall, our findings revealed physiological and transcriptomic characteristics of the Ahuehuete under waterlogging conditions, and provided new insights to waterlogging adaptation in woody gymnosperm species.

## 1. Introduction

*Taxodium mucronatum* Ten., also called Ahuehuete, sabino, or Montezuma bald cypress, is a riparian tree species of significant ecological, cultural, and economic importance [1,2]. The term “Ahuehuete” originates from the Nahuatl word āhuēhuētl, and its etymology can be interpreted as “upright drum in water” or “old man of the water”, alluding to the typical habitat in moist environments [1]. Like other *Taxodium* species, such as baldcypress (*T. distichum* var. *distichum* (L.) Rich.) and *T. ascendens* Brongn., the Ahuehuete demonstrates remarkable tolerance to prolonged flooding [3,4]. It was introduced to southeastern China in the last century for ecological enhancement and ornamental purposes. Subsequent evaluations have also confirmed their adaptability to this region, leading to their widespread application in coastal shelterbelt establishment and wetland ecological restoration [5,6]. Previous studies on *Taxodium* species, such as *T. distichum* and *Taxodium* hybrid ‘Zhongshanshan’, have identified several important physiological and transcriptional adaptation mechanisms, including aerenchyma formation, photosynthesis adjustments, organic acid metabolism, inhibition of abscisic acid (ABA) and jasmonic acid (JA) biosynthesis, and activation of specific pathways like glycolysis and fermentation [7,8,9,10,11,12,13,14]. However, the physiological and molecular adaptation mechanisms of the Ahuehuete to waterlogged environments remain poorly understood. In particular, it remains unclear whether this species shares these mechanisms entirely or has evolved distinct regulatory strategies to cope with its specific riparian habitats.

Water is fundamental to the survival of plants, but excessive water, such as waterlogging or flooding, impedes gaseous exchange between the soil and the atmosphere, and reduces the oxygen supply to plant roots, resulting in hypoxic or anoxic situations [15,16]. Many plants possess the ability to cope with short-term flooding stress under natural conditions, and likely harbor evolutionarily conserved acute flooding stress-related regulatory mechanisms [3]. In particular, plants synthesize a diverse array of osmoprotectants, including proline, soluble sugars, and soluble proteins, in response to osmotic stress induced by waterlogging [16,17,18]. The activation of the antioxidant enzyme system is another important approach to cope with waterlogging-induced ROS generation [19,20]. In *Styrax japonica* Sieb. et Zucc., seedlings exhibited compensatory upregulation of catalase (CAT) and peroxidase (POD) activities under waterlogging and partial submergence conditions [21]. Similarly, the activity of superoxide dismutase (SOD) and CAT increased in *Yulania stellata* (Siebold & Zucc.) N.H. Xia at the early stages of flooding stress [22].

Notably, only highly tolerant plant species can endure prolonged inundation, such species have developed specialized regulatory mechanisms in response to persistent waterlogging stress. For example, Giant reed (*Arundo donax* L.) has developed adaptive mechanisms to waterlogging stress, which include enhancing the activity of antioxidant enzymes to reduce oxidative damage, alongside employing osmoprotectants and aquaporins to regulate water homeostasis [23]. In baldcypress, the activation of the mitogen-activated protein kinase (MAPK) signaling pathway, antioxidant systems, immune systems, and glycolytic pathways are conserved mechanisms that respond to flooding stress [3].

MAPKs represent a class of highly conserved signaling transduction modules that play critical roles in numerous signal transduction processes through MAPK cascade pathways [24]. A typical MAPK cascade consists of three sequentially acting kinases: MAPK kinase kinase (MAPKKK, also known as MAP3K or MEKK), MAPK kinase (MAPKK, MAP2K, MKK, or MEK), and MAPK (also referred to as MPK) [25,26]. They are involved in the biosynthesis and/or signal transduction pathways of several stress-related phytohormones (e.g., ethylene, ABA, JA), consequently mediating essential functions within plant abiotic stress response [27,28,29].

In this study, we examined the dynamics of physiological characteristics (osmoprotectants, antioxidant enzyme system, malondialdehyde (MDA)) of the Ahuehuete leaves under waterlogging condition. Subsequently, temporal transcriptomes were performed to identify key regulatory pathways and hub genes involved in waterlogging adaptation through comparative analysis and weighted correlation network analysis (WGCNA). On this basis, this study provides a new insight to adaptive responses to waterlogging stress at the physiological and molecular levels.

## 2. Results

### 2.1. Physiological Response of the Ahuehuete Leaves to Waterlogging

**Figure 1:**
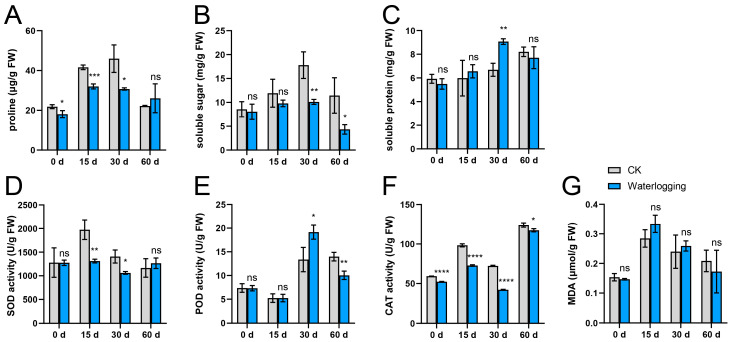
Comparative analysis of physiological indexes of the ahuehuete leaves under well-watered (CK) and waterlogging conditions. (**A**) Proline content. (**B**) Soluble sugar content. (**C**) Soluble protein content. (**D**) Superoxide dismutase (SOD) activity. (**E**) Peroxidase (POD) activity. (**F**) Catalase (CAT) activity. (**G**) Malondialdehyde (MDA) content. Sample size: n = 3. Asterisks indicate statistical differences at the *p* < 0.05 (*), 0.01 (**), 0.001 (***), and 0.0001 (****) levels. ns: not significant.

During the waterlogging treatment, the proline and soluble sugar contents exhibited an initial increase over the first 30 days, followed by a decline at 60 d. Throughout the experimental period, both parameters remained consistently lower than those observed in the control (CK) group (Figure 1A,B). In contrast to the CK group, the waterlogging treatment did not result in a significant difference in soluble protein content, with the exception of the measurement taken at 30 d (Figure 1C). Regarding antioxidant enzyme system, SOD activity showed relatively stable levels throughout the waterlogging treatment (Figure 1D). POD activity initially increased and subsequently decreased, reaching its peak at 30 d. Notably, the differences between the waterlogging and CK groups did not emerge until 30 d after the initiation of the experiment (Figure 1E). CAT activity exhibited a fluctuating upward trend throughout the experimental period, peaking at 60 d, with the waterlogging treatment consistently remaining lower than that of the CK group (Figure 1F). In addition, MDA content exhibited an initial increase followed by a subsequent decrease, with no significant difference observed between the waterlogging group and the CK group (Figure 1G).

### 2.2. Overview of Transcriptome Analysis

Transcriptomic samples were sequenced with three biological replicates per stage, yielding total raw reads ranging from 38,478,898 to 49,461,356 per sample (Table S1). After data filtering, between 38,342,710 and 49,071,202 clean reads were retained, with Q20 and Q30 values exceeding 98.25% and 94.90%, respectively, indicating high sequencing quality (Table S1). Subsequently, these clean reads were subjected to de novo assembly, generating a total of 101,585 unigenes (Table S2). Moreover, the length of the unigenes ranged from 201 bp to 23,773 bp, with an N50 length of 1703 bp (Table S2). Principal component analysis (PCA) demonstrated a clear separation between four different waterlogging stages, along with a strong correlation among three replicates at each stage, reflecting the high repeatability (Figure S1A). Clustering analysis also revealed that transcriptomic samples at D0 were separated from those at waterlogging conditions (D15, D30, and D60) (Figure S1B).

### 2.3. Analysis of Differentially Expressed Genes (DEGs) in the Ahuehuete Leaves Under Waterlogging Conditions

**Figure 2:**
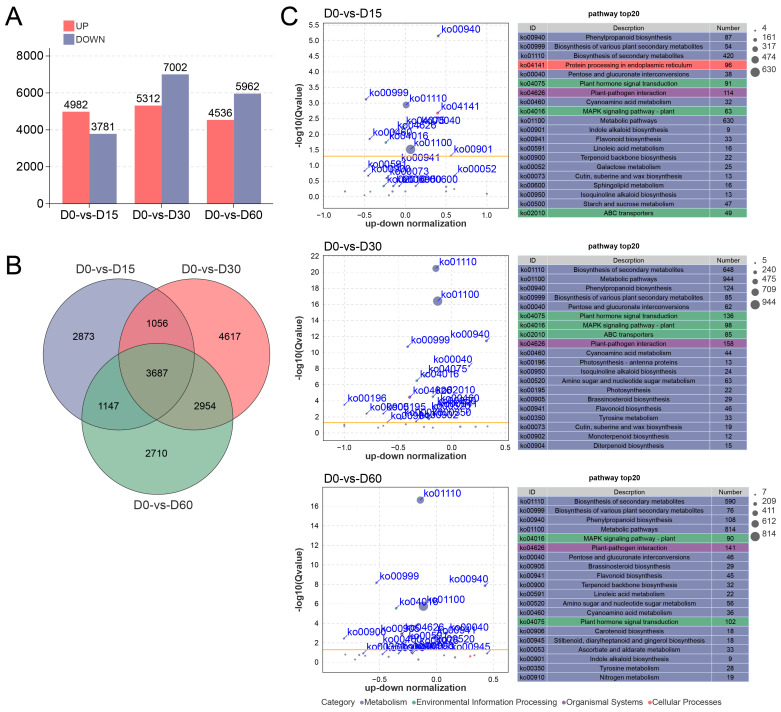
Transcriptome sequencing analysis of the Ahuehuete leaves in response to waterlogging condition. (**A**) Statistic analysis of up-/down-regulated DEGs. (**B**) Venn diagram of DEGs. (**C**) KEGG enrichment analysis of DEGs reveals the top 20 significantly enriched pathways.

A total of 4982 up- and 3781 down-regulated DEGs were identified in the D0 versus D15 comparison. In the comparison of D0 versus D30, 5312 DEGs were preferentially expressed at D0, whereas 7002 DEGs were preferentially expressed at D30. The D0 versus D60 comparison yielded 4536 up- and 5962 down-regulated DEGs (Figure 2A). The Venn diagram revealed that a total of 3687 DEGs were expressed in all comparisons throughout the waterlogging process, while 2873, 4617, and 2710 DEGs were exclusively identified in the comparisons of D0 versus D15, D0 versus D30, and D0 versus D60, respectively (Figure 2B).

Subsequently, a GO enrichment analysis was conducted to explore the biological functions of DEGs involved in waterlogging response. In the D0 versus D15 comparison, DEGs were significantly enriched in terms related to oxidoreductase activity (GO:0016491), antioxidant activity (GO:0016209), peroxidase activity (GO:0004601), etc. DEGs in the D0 versus D30 comparison were mainly enriched oxidoreductase activity (GO:0016491) and DNA-binding transcription factor activity (GO:0003700). For the D0 versus D60 comparison, DEGs were involved in oxidoreductase activity (GO:0016491), antioxidant activity (GO:0016209), ATP hydrolysis activity (GO:0016887), etc. (Figure S2).

KEGG enrichment analysis revealed that pathways pertaining to the Metabolism category constituted the highest proportion, followed by those associated with Environmental Information Processing category (Figure 2C). Enriched pathways consistently identified across all comparative groups included Phenylpropanoid biosynthesis (ko00940), Flavonoid biosynthesis (ko00941), Plant hormone signal transduction (ko04075), and MAPK signaling pathway-plant (ko04016) (Figure 2C), indicating that these pathways may play a crucial role in the adaptation of the Ahuehuete to waterlogging. In addition, after 15 and 30 days of waterlogging stress, the ABC transporter (ko02010) was significantly enriched, and the Nitrogen metabolism (ko00910) was significantly enriched after 60 days (Figure 2C). These results implied that long-term waterlogging stress might induce nitrogen deficiency and alter gene expression of nitrogen metabolism-related pathways.

### 2.4. WGCNA Identified Key Module and Hub Genes Associated with Waterlogging Adaptation

**Figure 3:**
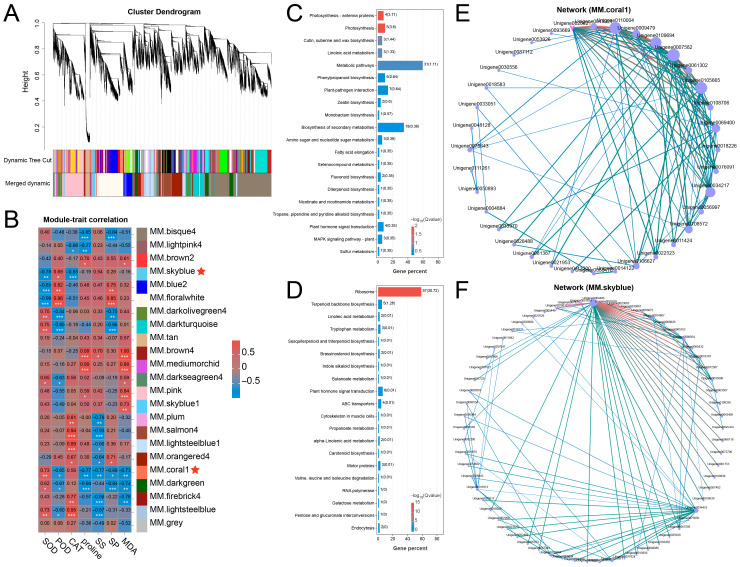
WGCNA identifies key modules and candidate genes responsible for waterlogging adaptation. (**A**) Identification of modules. (**B**) Correlation analysis reveals key modules related to waterlogging adaptation. Asterisks indicate significance at *p* < 0.05 (*), *p* < 0.01 (**), and *p* < 0.001 (***). (**C**,**D**) KEGG analysis of genes from the coral1 (**C**) and skyblue (**D**) modules. (**E**,**F**) Co-expression network derived from the coral1 (**E**) and skyblue (**F**) module identifies hub genes associated with waterlogging adaptation.

WGCNA was performed to identify gene clusters co-expressed in waterlogging conditions, setting the power value at 19 and the mean connectivity of 418.64688 (Figure S3A,B). A total of 19,044 DEGs were grouped into 23 modules, each comprising between 1 and 4909 genes (Figure 3A and Figure S3C). The module–trait correlation analysis demonstrated that MM.coral1 was significantly correlated with SOD, POD, proline, SS, SP, and MDA, and MM.skyblue was significantly correlated with SOD, POD, and CAT (Figure 3B). In addition, KEGG analysis showed that the MM.coral module harbored genes linked to various pathways, including Phenylpropanoid biosynthesis, Flavonoid biosynthesis, Plant hormone signal transduction, and MAPK signaling pathway-plant (Figure 3C). Genes from the MM.skyblue module were enriched in Plant hormone signal transduction, etc. (Figure 3D). Furthermore, co-expression network analysis identified several hub genes within distinct key modules. Specifically, the MM.coral1 module contained *ERF017* (Unigene0108706), *BGAL8* (Unigene0069400), and *Histone H1.2* (Unigene0007582) (Figure 3E), whereas the MM.skyblue module included *MYB4* (Unigene0007825) and *MYB5* (Unigene0020816) (Figure 3F). These results imply that genes in MM.coral1 and MM.skyblue might play important roles in adaptation of the Ahuehuete to waterlogging.

### 2.5. Regulatory Road Maps for Plant Hormone Signal Transduction and MAPK Signaling Pathway

Comparative transcriptomic analysis and WGCNA have revealed that Plant hormone signal transduction (ko04075) and MAPK signaling pathway-plant (ko04016) might play important roles in waterlogging adaptation. Therefore, we further focused on the road maps of these pathways and identified key DEGs.

**Figure 4:**
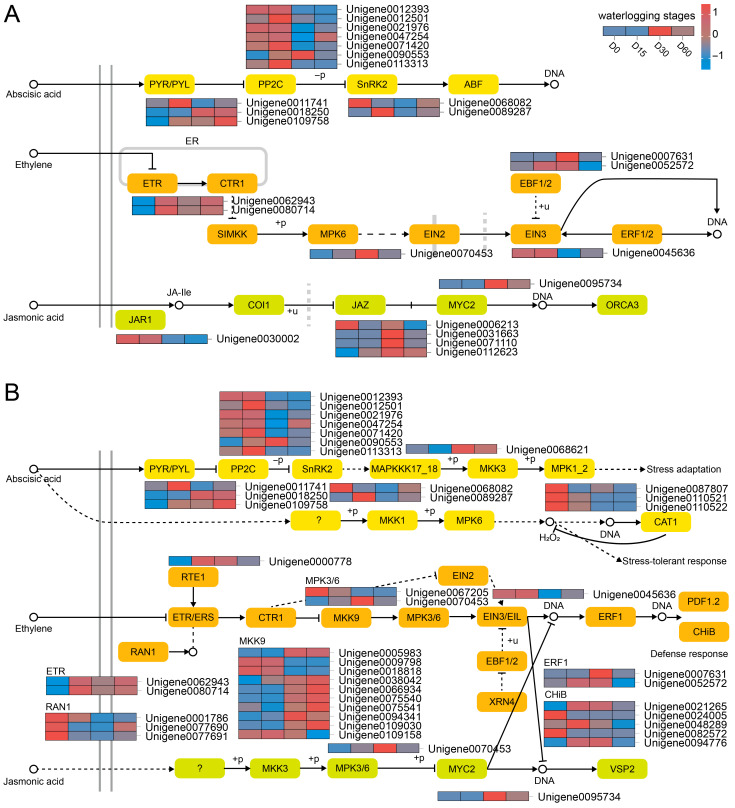
Regulatory road maps for plant hormone signal transduction (**A**) and MAPK signaling pathway (**B**).

Consequently, our analysis identified a subset of DEGs implicated in the signal transduction pathways of ABA, ethylene (ET), and JA (Figure 4A). ABA signal transduction-related DEGs included *PYL* (Unigene0011741, Unigene0018250, and Unigene0109758), and *PP2C* (e.g., Unigene0012393), *SnRK2* (Unigene0068082, Unigene0089287). *ETR* (Unigene0062943, Unigene0080714), *MPK6* (Unigene0070453), *EIN3* (Unigene0045636), and *EBF1/2* (Unigene0007631, Unigene0052572) were involved in ET signal transduction. In addition, *JAR1* (Unigene0030002), *JAZ* (e.g., Unigene0006213), and *MYC2* (Unigene0095734) were associated with JA signal transduction.

Furthermore, MAPK signal cascade related to ABA involved *MAPKKK17_18* (Unigene0068621), and *MPK1_2* (Unigene0087807, etc.) (Figure 4B). As for ET-associated MAPK signal pathway, the related DEGs were *MPK3/6* (Unigene0067205, Unigene0070453), *MKK9* (e.g., Unigene0005983), *RAN1* (Unigene0001786, etc.), *ERF1* (Unigene0007631, Unigene0052572), and *CHiB* (e.g., Unigene0021265). Additionally, *MPK6* (Unigene0070453) participated in JA-related MAPK signal pathway.

### 2.6. RT-qPCR Verification of Key Genes Associated with Waterlogging Adaptation

**Figure 5:**
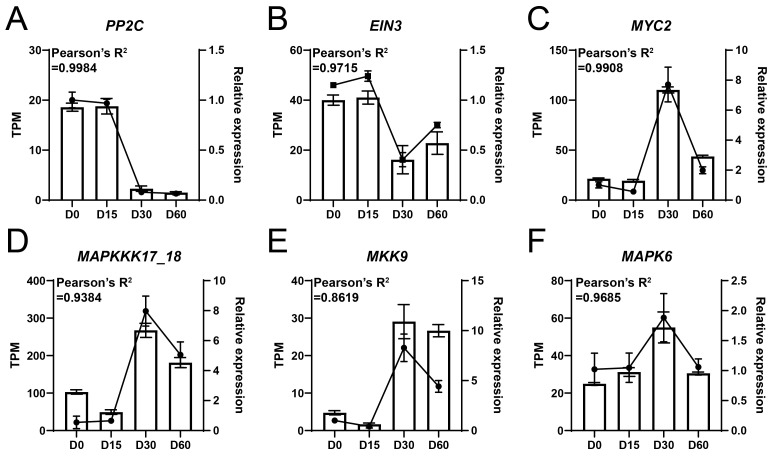
RT-qPCR assay of key genes associated with waterlogging adaptation in the Ahuehuete. (**A**) *PP2C*. (**B**) *EIN3*. (**C**) *MYC2*. (**D**) *MAPKKK17_18*. (**E**) *MKK9*. (**F**) *MAPK6*. Bars represent TPM values (from RNA-seq), while dotted lines indicate relative expression levels (from RT-qPCR).

RT-qPCR assay was performed to assess the reliability of the high-throughput RNA-sequencing. The results showed that *PP2C* and *EIN3* were highly expressed at D0 and D15, while their expression levels decreased at D30 and D60 (Figure 5A,B). In addition, the expression levels of the *MYC2*, *MAPKKK17_18*, *MKK9*, and *MAPK6* genes were relatively low at D0 and D15, followed by an increase at D30 and a decrease at D60 (Figure 5C–F). Importantly, the correlation value between the results of RT-qPCR and RNA-sequencing exceeded 0.8619, suggesting the high reliability of RNA-seq data (Figure 5).

## 3. Discussion

Waterlogging stress represents one of the major abiotic constraints limiting global agricultural productivity and destabilizing natural ecosystems [30]. It induces soil hypoxia even anoxia, which in turn triggers a series of complex physiological, biochemical, and molecular cascades that severely impair plant survival, growth, and reproduction [31]. Waterlogging stress disrupts the electron transport chains in both mitochondria and chloroplasts, leading to the excessive accumulation of reactive oxygen species (ROS) such as superoxide anion ($O_{2}^{-}$) and hydrogen peroxide (H_2_O_2_), thereby inducing oxidative damage [32,33]. Compared to the control group, the leaves of tomato plants subjected to waterlogging stress showed a significant increase in the content of oxidative parameters (H_2_O_2_ and MDA) [34]. Conversely, our present study demonstrated no significant difference in MDA content between the Ahuehuete leaves subjected to waterlogging and those under well-watered conditions (Figure 1G). This indicates that the waterlogging did not cause damage to the Ahuehuete, or alternatively, that this species has developed an adaptation to waterlogging conditions.

To mitigate oxidative damage, plants have developed intricate antioxidant enzyme system in response to waterlogging stress, involving SOD, POD, CAT, etc. [35]. Under waterlogging conditions, the activities of CAT and SOD were enhanced in pigeonpea (*Cajanus cajan* (L.) Millsp.) roots. Notably, waterlogging-resistant lines exhibited significantly higher enzymatic activities compared to waterlogging-sensitive lines [36]. In *Yulania stellata* (Siebold & Zucc.) N.H. Xia, the activity of SOD and CAT was also increased by flooding stress [22]. Similarly, seedlings of *Styrax japonica* Sieb. et Zucc. exhibited compensatory increase in CAT and POD activities under waterlogging and partial submergence conditions [21]. As a waterlogging-tolerant species, the activities of antioxidant enzymes in the Ahuehuete under waterlogging condition were similar to those of well-watered condition. Moreover, at most time points, the enzymatic activity under waterlogging conditions was lower than that of the control (Figure 1D–F). These findings suggest that the Ahuehuete is well acclimated to waterlogging, necessitating only basal levels of antioxidant enzyme activity to cope with environmental stress. This contrasts with observations in waterlogging-sensitive species. For instance, in waterlogging-sensitive maize genotype K12, the POD activity is significantly inhibited by long-term waterlogging [37]. Similarly, in pigeon pea (*Cajanus cajan* L.), SOD and CAT activities continuously increase in the tolerant genotype ICPL 84023 as waterlogging persists, whereas they decline progressively in susceptible genotype ICP 7035 [38]. In the present study, antioxidant enzyme system of the Ahuehuete was still active after exposure to long-term waterlogging (Figure 1D–F). It may thus be inferred that the enhancement of antioxidant enzyme activities contributes to improved plant tolerance under long-term waterlogging conditions.

Waterlogging exerts a significant influence on the osmotic regulation processes in plants. A key physiological adaptation mechanism to short-term waterlogging stress involves the accumulation of osmoregulatory substances—including soluble proteins, sugars, and free proline [39,40]. After being subjected to waterlogging stress for 10 d, cucumber plants accumulated significantly higher levels of proline and glucose compared to those under non-waterlogged conditions [41]. In contrast, the levels of total and non-reducing sugars declined in mung bean (*Vigna radiata*) plants when subjected to an eight-day waterlogging treatment [42]. We observed an initial increase followed by the decrease in osmoprotectants during waterlogging conditions (Figure 1A–C). These results suggest that the Ahuehuete underwent dynamic adjustments in osmoprotectant levels in response to both short- and long-term waterlogging stress. The decrease in proline and soluble sugar content relative to the control may reflect a lower demand for osmotic adjustment under waterlogging conditions in this waterlogging-tolerant species, whereas the transient increase in soluble protein at D30 could represent a specific short-term adaptive mechanism.

High-resolution transcriptomic technology has been widely used to provide molecular insights into the genetic basis of plant responses to waterlogging or flooding stress [43,44,45,46]. To explore the molecular mechanism of the Ahuehuete in adaptation to waterlogging stress, we constructed time-order transcriptomic libraries for high-throughput sequencing. Comparative analysis and WGCNA identified key metabolic pathway, including Plant hormone signal transduction (ko04075), and MAPK signaling pathway-plant (ko04016) (Figure 2C and Figure 4). This phenomenon was consistent with the results observed in *Actinidia valvata* Dunn [46], barley [47], *Sesamum indicum* L. [48], and *Secale cereale* L. [49]. Phytohormones perform critical regulatory functions across a spectrum of molecular, morphological, biochemical, anatomical, and signaling processes that are essential for plant survival under oxygen-deprived stress conditions [50,51]. Under waterlogging conditions, 1-aminocyclopropane-1-carboxylic acid (ACC) is produced under the catalysis of ACC synthase (ACS), and then transferred from plant root system to the aerobic region, resulting in the production of ET by the catalysis of ACC oxidase (ACO) [52]. In the study on sweet potato, it was observed that the waterlogging-tolerant variety exhibited higher expression levels of genes associated with ET synthesis and signal transduction (*ETR1*, *ERS1*, and *EIN2*) under low-oxygen conditions [53]. Similarly, *ETR*, *EIN3*, and *EBF1/2* were differentially expressed in the Ahuehuete leaves during waterlogging condition (Figure 4). In particular, *MKK9-MPK3/MPK6* modules act downstream of the ET receptors, to control the key transcription factor EIN3 and downstream transcription cascades [54]. In addition to ET, other plant hormones such as ABA and JA also play critical roles in tolerance to waterlogging [55]. For example, Exogenous ABA application positively regulated the JA content in adventitious roots of *Cleistocalyx operculatus* and *Syzygium jambos* under long-term waterlogging, enhancing tolerance to stress [56]. In *Actinidia deliciosa*, a novel ethylene-responsive transcription factor named AdRAP2.3 could be induced by MeJA, ACC, ABA, waterlogging, etc. Overexpression of AdRAP2.3 in transgenic tobacco improved resistance to waterlogging through regulation of genes related to pyruvate decarboxylase (PDC) and alcohol dehydrogenase (ADH) [57]. We also identified several DEGs implicated in the signal transduction pathways of ABA and JA, and MAPK signal cascades, including *PYL*, *PP2C*, *SnRK2*, *JAR1*, *JAZ*, *MYC2*, *MAPKKK17_18*, *MPK1_2* (Figure 4). These results imply that MAPK-dependent hormonal signaling pathway play an important role in adaptation of the Ahuehuete to waterlogging. Given the complexity of the hormonal regulatory network under flooding stress, the significance of hormones such as auxin and GA should not be overlooked and warrants further investigation, even though their signal transduction is not regulated by the MAPK cascade. Furthermore, the physiological and molecular changes induced by waterlogging are considerably more complex than previously discussed.

## 4. Materials and Methods

### 4.1. Plant Materials and Cultivation Conditions

The experimental materials were two-year-old container-grown seedlings of *T. mucronatum*, with an average plant height of approximately 100 cm. The seedlings were transplanted in the spring of 2024 into plastic nursery pots measuring 15 cm in height and 20 cm in diameter, with one seedling per pot. Garden soil (texture: clay; pH 5.13; N: 0.801 g/kg; P: 0.38 g/kg; K: 86.42 g/kg) was used as the growing medium.

The experimental site was located at the Baima Teaching and Research Base of Nanjing Forestry University in Lishui District, Nanjing City, Jiangsu Province (119°168′ E, 31°609′ N). This region experiences a subtropical monsoon climate, characterized by hot and rainy summers along with dry and cold winters. The mean annual temperature is approximately 15.4 °C, and the mean annual precipitation reaches 1204.3 mm. The experiment was initiated in July, during which the average temperature at the site was about 32 °C over the two-month experimental period. The seedling planting area was subjected to shading treatment using 16 needle shade net. Light intensity monitoring using a Temperature, humidity and light recorder DJL-18-G (Tuopuyunnong, Hangzhou, China) showed a shading rate of approximately 80% (15,930–17,458 Lux).

### 4.2. Waterlogging Treatment and Sample Collection

**Figure 6:**
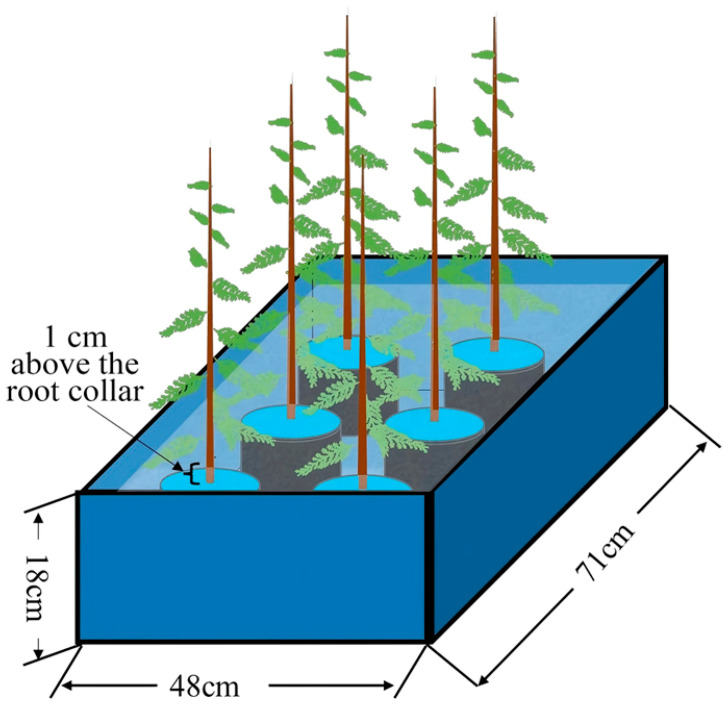
Schematic diagram of waterlogging treatment.

In late July 2024, robust seedlings with largely uniform growth were selected for waterlogging stress treatment. Two experimental conditions were established: waterlogged and control, with three replicates per condition. Each replicate consisted of 20 seedlings. Control (CK) treatment: Seedlings received conventional irrigation management, with the substrate moisture maintained at approximately 75% of field capacity. Waterlogging treatment: The waterlogging process was conducted using a double-pot method. Seedlings were placed in large plastic containers measuring 71 cm (length) × 48 cm (width) × 18 cm (height), with tap water filled to a level 1 cm above the root collar of the seedlings (Figure 6). The water level within the containers was monitored daily, and supplementary water was added as necessary to maintain the predetermined flooding depth. Importantly, several small holes were drilled into the side walls of the outer pot slightly above the intended water level, allowing excess water to drain once it exceeded the preset height. At 0, 15, 30, 60 d, fresh leaves were collected from three randomly selected seedlings per replicate and rapidly frozen using liquid nitrogen, and subsequently stored at −80 °C for future use.

### 4.3. Determination of MDA Content

The MDA content was quantified in accordance with an established protocol [58]. Briefly, leaf tissue samples (0.5 g fresh weight) were homogenized in 6 mL of 0.1 M phosphate buffer (pH 7.0). The resulting homogenate was transferred into a 10 mL centrifuge tube and centrifuged at 5500 rpm for 15 min at 4 °C. The supernatant was then collected and stored at 4 °C for subsequent analysis. For the assay, 1.0 mL of the supernatant was combined with 3 mL of 10% (*w*/*v*) trichloroacetic acid (TCA) and 1 mL of 0.6% (*w*/*v*) thiobarbituric acid (TBA), and the mixture was boiled for 15 min. After cooling to room temperature, the solution was centrifuged again under the same conditions. The absorbance of the final supernatant was measured spectrophotometrically at 450 nm, 532 nm, and 660 nm to quantify the MDA concentration using a PerkinElmer Lambda 950 spectrophotometer (PerkinElmer, Hopkinton, MA, USA).

### 4.4. Antioxidant Enzyme Activity Measurements

Superoxide dismutase (SOD) activity was quantified spectrophotometrically based on the inhibition of nitro blue tetrazolium (NBT) photochemical reduction, following the method described by Chen et al. [58]. The reaction mixture consisted of 0.1 M phosphate buffer (pH 7.8), 130 mM methionine, 750 µM NBT, 100 µM EDTA-Na2, and 20 µM riboflavin. Prior to absorbance measurement at 560 nm, 0.1 mL of enzyme supernatant was introduced into the assay system.

Peroxidase (POD) activity was determined using the guaiacol oxidation method. A mixture was prepared by adding 0.1 mL of supernatant to 3.8 mL of 0.3% (*w*/*v*) guaiacol and 0.1 mL of 3% (*v*/*v*) hydrogen peroxide, after which the absorbance was monitored at 470 nm.

Catalase (CAT) activity was assessed according to an established protocol wherein 0.2 mL of enzyme extract was reacted with the mixed solution (containing 1.5 mL of 0.1 M phosphate buffer (pH 7.8), 1 mL of ddH_2_O, and 0.3 mL of 0.1 M hydrogen peroxide solution). The decrease in absorbance at 240 nm was recorded over a 30 s period to evaluate catalytic activity.

### 4.5. Quantification of Soluble Sugar, Soluble Protein, and Proline

The soluble sugar content was determined using the anthrone–sulfuric acid method [59]. Leaf tissue samples (0.2 g FW) were ground and subjected to two consecutive heating cycles in a 100 °C water bath, followed by centrifugation at 3500× *g* for 10 min. The resulting supernatant was collected for subsequent analyses.

The soluble protein content was quantified using the Bradford method [58]. Specifically, 1.0 mL of the supernatant was combined with 5.0 mL of Coomassie Brilliant Blue solution (0.1 g/L), and the absorbance was measured at a wavelength of 595 nm.

Proline content was analyzed according to a modified spectrophotometric protocol [60]. Briefly, fresh leaf tissues (0.25 g) were ground and then extracted in 5 mL of 3% sulfosalicylic acid solution using a 100 °C water bath for 30 min. The extract was then mixed with an equal volume of glacial acetic acid and acidic ninhydrin reagent, and incubated in a boiling water bath for 30 min. After cooling to room temperature, 4 mL of toluene was added, and the mixture was vigorously vortexed for 60 s. Following a 10 min phase-separation period, the upper toluene layer containing the chromophore was collected, and its absorbance was measured at 520 nm for proline concentration calculation. All physiological index assays included three biological replicates.

### 4.6. RNA Extraction, Library Construction, and Transcriptome Sequencing

Total RNA extraction, library preparation, and RNA sequencing were performed in accordance with established protocols from our previous study [59]. Briefly, total RNA was isolated from the collected tissues using a Trizol Reagent Kit (Invitrogen, Carlsbad, CA, USA) according to the manufacturer’s instructions. RNA quality was then assessed with an Agilent 2100 Bioanalyzer (Agilent Technologies, Palo Alto, CA, USA) and verified through RNase-free agarose gel electrophoresis. Following extraction, eukaryotic mRNA was enriched using Oligo (dT) magnetic beads and fragmented into short segments. cDNA synthesis was carried out using the NEBNext Ultra RNA Library Prep Kit for Illumina (New England Biolabs, Beverly, MA, USA). The resulting fragments underwent end repair, adenylation, and adapter ligation, followed by purification with 1.0× AMPure XP Beads. cDNA libraries were then constructed via polymerase chain reaction (PCR) amplification. Finally, transcriptome sequencing was conducted on the Illumina NovaSeq 6000 platform (Illumina, Inc., San Diego, CA, USA).

### 4.7. Transcriptomic Data Analysis

Raw sequencing reads were processed using Fastp (-q 20 -u 50 -N 15 -l 50, version 0.18.0) [61] to generate high-quality clean reads. These clean reads were then aligned to a ribosomal RNA (rRNA) reference database using Bowtie2 (--local, version 2.2.8) [62]. Reads mapped to rRNA were removed, and the remaining high-quality reads were de novo assembled into transcripts using StringTie (-f 0.3, v1.3.1) [63]. Gene abundance was quantified via the FPKM (fragments per kilobase of transcript per million mapped reads) method with RSEM software (default, version 1.3.3) [64].

Differentially expressed genes (DEGs) were identified based on a binomial distribution model implemented in DEGseq (default, v1.36.1), with thresholds set at |log2(Fold Change)| ≥ 1 and a Q value ≤ 0.05 [65]. Subsequently, Gene Ontology (GO) and Kyoto Encyclopedia of Genes and Genomes (KEGG) pathway enrichment analyses were conducted to elucidate the biological functions of the DEGs. Significantly enriched terms or pathways were defined using a Q value threshold of ≤0.05.

The gene set employed in the weighted correlation network analysis (WGCNA) was constructed from transcriptomic data, retaining all DEGs identified across any comparison group. WGCNA was implemented using the “WGCNA” package (version 1.73) in R software (version 4.3.1) [66]. The soft thresholding power was determined based on scale-free topology fit index analysis. A power value of 19 was selected, which satisfied the scale-free topology criterion with a fit index exceeding 0.800985 while preserving a high mean connectivity (418.64688) (Figure S3A,B). Module detection was conducted using default parameters, with the maximum module size and the minimum number of genes per module set to 20 and 50, respectively. In addition, the correlation between physiological traits and modules was generated based on Pearson correlation coefficients calculated between module membership values and trait data, thus identifying key modules. Subsequently, hub genes with high connectivity were identified in key modules.

### 4.8. RT-qPCR Validation

cDNA was synthesized from the total RNA using the *Evo M-MLV* RT Kit with gDNA Clean for qPCR (Accurate Biotechnology (Hunan) Co., Ltd., Changsha, China). Then, RT-qPCR was performed using the SYBR Green Premix Pro Taq HS qPCR Kit (Accurate Biotechnology (Hunan) Co., Ltd., Changsha, China) on the ABI StepOnePlus Real-Time PCR system (Applied Biosystems Inc., Foster City, CA, USA). All primers used in this study were listed in Table S3.

### 4.9. Statistical Analysis

Data visualization was conducted utilizing the online Omicsmart platform (http://www.omicsmart.com, accessed on 1 September 2025). Bar graphs were constructed using GraphPad Prism (Version 9.5.1; GraphPad Software, Boston, MA, USA). For statistical analysis, multiple unpaired *t* tests (*p* < 0.05) were performed with IBM SPSS Statistics 26 (International Business Machines Corporation, Armonk, NY, USA) to assess significant difference.

## 5. Conclusions

Based on the integrated physiological and transcriptomic analyses, this study demonstrates that Ahuehuete employs a sophisticated adaptive strategy to waterlogging stress. Physiologically, it exhibits a dynamic response with initial accumulation of osmoregulatory substances (soluble sugar, soluble protein, and proline) coupled with modulated activities of antioxidant enzymes (SOD, POD, and CAT) to mitigate oxidative damage. Transcriptome profiling revealed a significant reprogramming of gene expression, highlighting the crucial involvement of phenylpropanoid and flavonoid biosynthesis, plant hormone signal transduction, and the MAPK signaling pathway. Weighted Gene Co-expression Network Analysis (WGCNA) identified key modules (coral1, skyblue) highly correlated with physiological traits and pinpointed several hub genes, including *ERF017* and *MYB4*. KEGG enrichment analysis of DEGs from key modules underlined the adaptive response by regulating ABA, ET, and JA signaling pathways, as well as MAPK cascades. Our findings provide a comprehensive molecular framework underlying Ahuehuete’s tolerance to waterlogging.

## Data Availability

The data are contained within this article.

## Supplementary Materials

The following supporting information can be downloaded at: https://www.mdpi.com/article/10.3390/plants14213295/s1, Figure S1: (A) Principal component analysis of the Ahuehuete leaf samples using transcriptome data averaged from three replicates. The first two principal components are shown. (B) Clustering analysis. Figure S2: GO enrichment analysis of DEGs involved in waterlogging response; Figure S3: Soft threshold selection for WGCNA. (A) Scale independence shows the relationship between the soft threshold power and the scale-free topology fit index. (B) Mean connectivity in the right panel indicates the relationship between the soft threshold power and mean connectivity. (C) Gene number for each module; Table S1: Statistical information of transcriptome libraries of the Ahuehuete leaves under waterlogging conditions; Table S2: Statistical analysis of assembly quality; Table S3: Primers used for RT-qPCR assay.
