# Supplementary material for: Physiological and Molecular Adaptation of the Ahuehuete (Taxodium mucronatum Ten.) to Waterlogging
Source: Plants (Basel). 2025 Oct 29;14(21):3295. doi: 10.3390/plants14213295 (PMC12610694; doi:10.3390/plants14213295)
Supplement: Supplementary file 1 [file plants-14-03295-s001.zip › plants-3892527-supplementary.pdf]

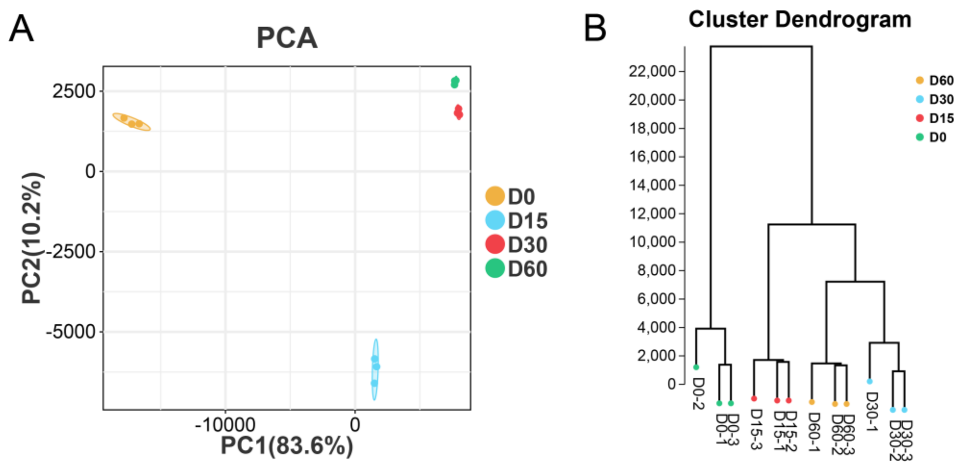

Figure S1. (A) Principal component analysis of the Ahuehuete leaf samples using transcriptome data averaged from three replicates. The first two principal components are shown. (B) Clustering analysis.

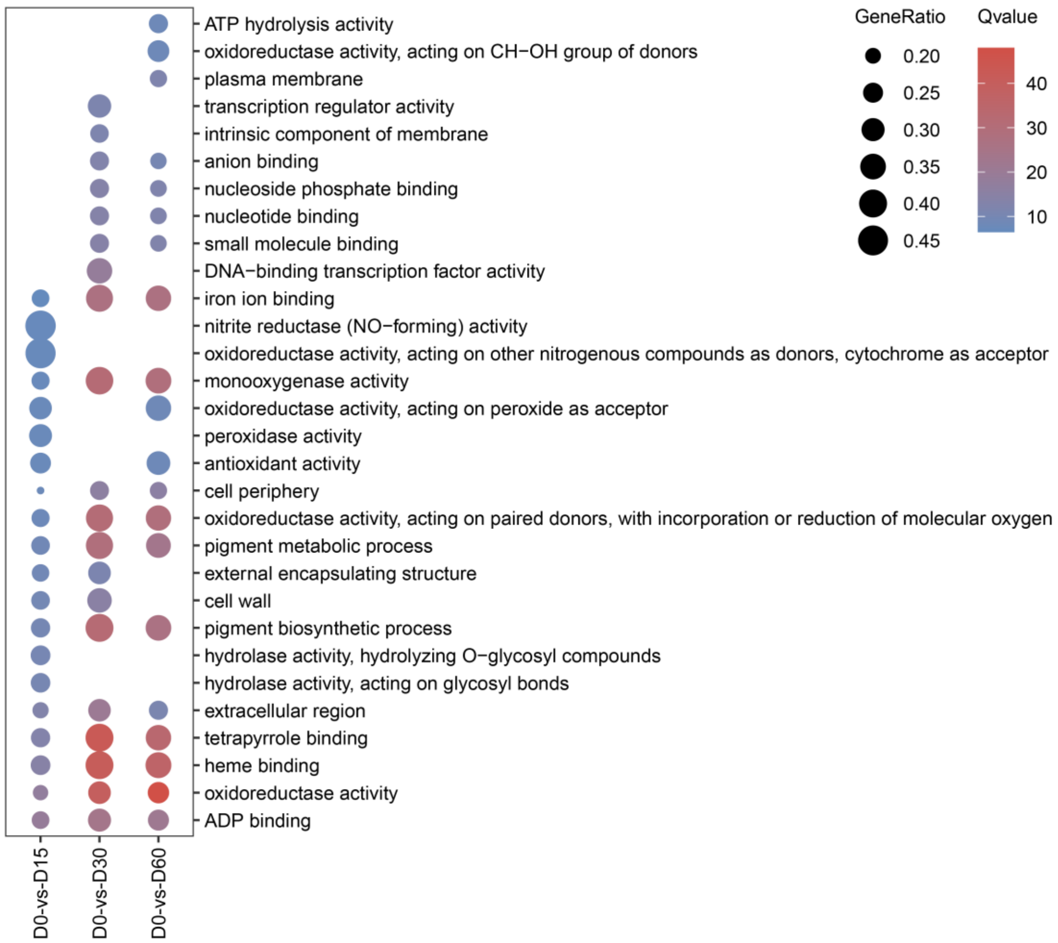

Figure S2. GO enrichment analysis of DEGs involved in waterlogging response.

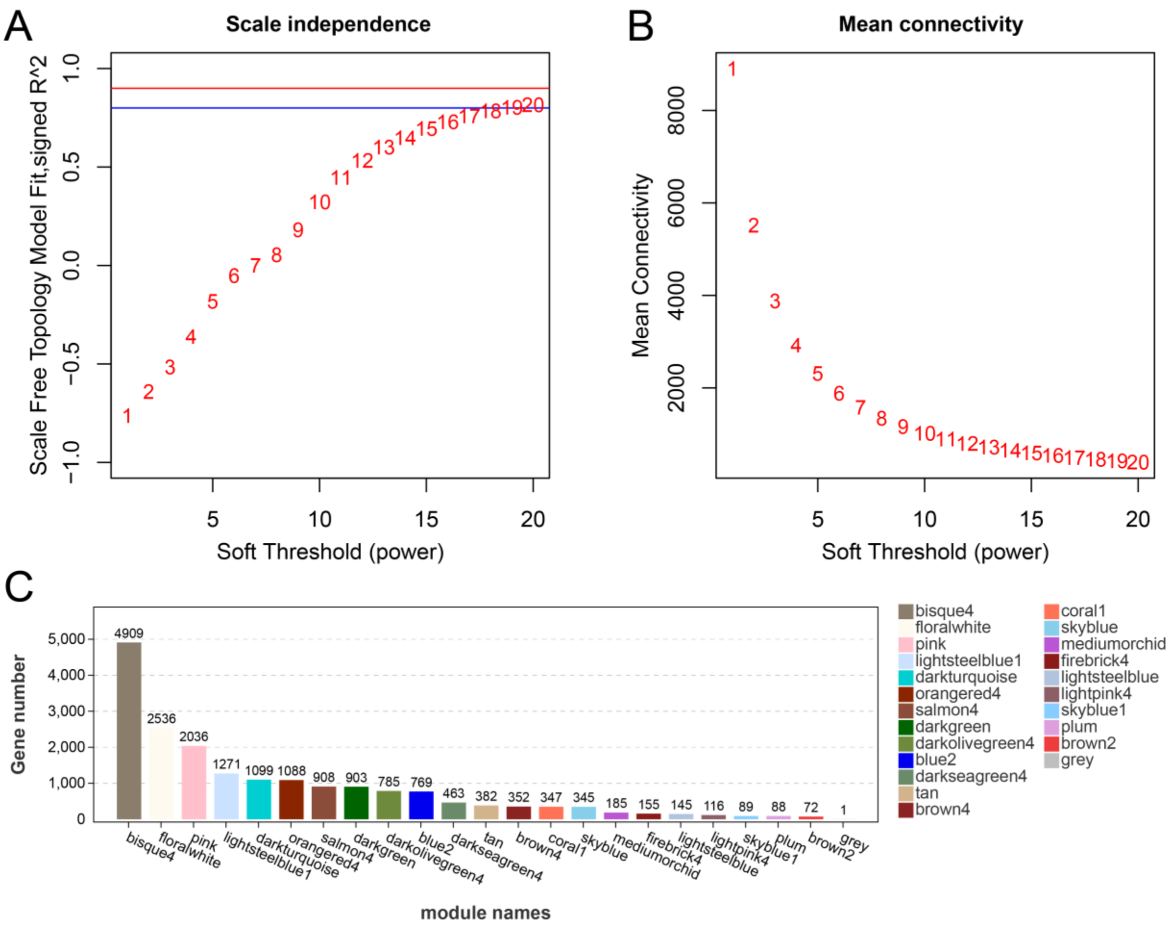

Figure S3. Soft threshold selection for WGCNA. (A) Scale independence shows the relationship between the soft threshold power and the scale-free topology fit index. (B) Mean connectivity in the right panel indicates the relationship between the soft threshold power and mean connectivity. (C) Gene number for each module.

Table S1 Statistical information of transcriptome libraries of the Ahuehuete leaves under waterlogging conditions

Sample	RawDatas	Adapter(%)	LowQuality(%)	polyA(%)	N(%)	CleanData(%)	Q20(%)	Q30(%)
D0-1	38478898	11248 (0.03%)	124628 (0.32%)	0 (0.00%)	312 (0.00%)	38342710 (99.65%)	98.78%	96.33%
D0-2	43156022	12044 (0.03%)	128264 (0.30%)	0 (0.00%)	358 (0.00%)	43015356 (99.67%)	98.78%	96.32%
D0-3	37255382	9864 (0.03%)	125952 (0.34%)	0 (0.00%)	336 (0.00%)	37119230 (99.63%)	98.78%	96.33%
D15-1	36101466	15376 (0.04%)	150088 (0.42%)	0 (0.00%)	616 (0.00%)	35935386 (99.54%)	98.66%	95.85%
D15-2	39395440	16396 (0.04%)	175826 (0.45%)	0 (0.00%)	50 (0.00%)	39203168 (99.51%)	98.51%	95.45%
D15-3	40589862	25116 (0.06%)	202270 (0.50%)	0 (0.00%)	606 (0.00%)	40361870 (99.44%)	98.53%	95.59%
D30-1	41530088	16858 (0.04%)	226670 (0.55%)	0 (0.00%)	602 (0.00%)	41285958 (99.41%)	98.48%	95.46%
D30-2	41675022	17080 (0.04%)	229800 (0.55%)	0 (0.00%)	656 (0.00%)	41427486 (99.41%)	98.51%	95.51%
D30-3	38437516	14790 (0.04%)	179782 (0.47%)	0 (0.00%)	640 (0.00%)	38242304 (99.49%)	98.74%	96.13%
D60-1	37179086	16962 (0.05%)	232086 (0.62%)	0 (0.00%)	630 (0.00%)	36929408 (99.33%)	98.25%	94.90%
D60-2	36388336	11052 (0.03%)	147386 (0.41%)	0 (0.00%)	672 (0.00%)	36229226 (99.56%)	98.79%	96.30%
D60-3	49461356	21630 (0.04%)	367888 (0.74%)	0 (0.00%)	636 (0.00%)	49071202 (99.21%)	98.28%	95.15%

Table S2 Statistical analysis of assembly quality

Genes Number	GC percentage (%)	N50 number	N50 length (bp)	Max length (bp)	Min length (bp)	Average length (bp)	Total assembled bases (bp)
101585	39.1424	14931	1703	23773	201	924	93870861

Table S3 Primers used for RT-qPCR assay.

Gene name	Gene id	Forward primer (5'-3')	Reverse primer (5'-3')	Product size (bp)
PP2C	Unigene0012393	CCGTTTGTCGGACCCTGTAA	CTTCGAGGTGCTTCCCACTT	113
EIN3	Unigene0045636	TGGGAATGAGGATTGGTGGC	ACCCCTACCTTCCAGGACTT	102
MYC2	Unigene0095734	TGAGCCAGGAGACATTGCAG	TAGTACCCATCACCCCAGCA	136
MPK6	Unigene0070453	GCAGCCATTGATGTGTGGTC	AAGATCAGCCTCCGTTGGTG	138
MAPKKK17_18	Unigene0068621	AGAATGGGGTTGAGGTCTGC	TCCAATCCACGAAGGATGCC	139
MKK9	Unigene0005983	GAGGCTTCCTTTGCCGATCT	CCAACAATCCTCTTGCTGCG	119
